# A Novel Mutation in *SNX10* Gene Causes Malignant Infantile Osteopetrosis

**Published:** 2017

**Authors:** Akbar Amirfiroozy, Amir A. Hamidieh, Zahra Golchehre, Azim Rezamand, Mahin Yahyaei, Fatemeh Beiranvandi, Soheyla Amirfiroozy, Mohammad Keramatipour

**Affiliations:** 1. Department of Medical Genetics, Faculty of Medicine, Tehran University of Medical Sciences, Tehran, Iran; 2. Hematology-Oncology and Stem Cell Transplantation Research Center, Tehran University of Medical Sciences, Tehran, Iran; 3. Children’s Hospital, Tabriz University of Medical Sciences, Tabriz, Iran

**Keywords:** Iran, Mutation, Osteopetrosis, *SNX10*

## Abstract

**Background:**

Osteopetrosis is a group of genetically heterogonous diseases and the main feature of that is increased bone density due to osteoclast’s abnormality. It has three clinical forms based on inheritance pattern, severity and age of onset: the dominant benign form (ADO), the intermediate form (IRO) and the recessive severe form (ARO). One of the recently discovered genes for ARO form is *SNX10* that accounts for 4% of affected persons by this type.

**Methods:**

In this paper, a 15 years old girl affected by osteopetrosis has been analyzed for detecting causal mutation in known osteopetrosis genes. To get it done, amplified exons of the genes were sequenced and then were analyzed.

**Results:**

Direct sequencing of *SNX10* gene showed a homozygous c.43delG variant in the patient. Both healthy parents were heterozygous for this variant. In silico analysis revealed that this novel variant can be considered as the cause of disease in the patient.

**Conclusion:**

In this paper, a girl affected by osteopetrosis with a novel deletion in *SNX10* gene was reported.

## Introduction

Osteopetrosis is a group of genetically heterogeneous diseases that affects bones. It was first described by Albers-Schönberg, a century ago who named it “marble bone disease”. The disease results from abnormal bone remodeling due to osteoclast disorders. Osteoclasts are bone specific cells originated from hematopoietic lineage that reabsorb bone matrix, so in correlation with osteoblasts, as bone-forming cells, remodel the bone. The abnormal bone formation in micro structure level leads the bone to be fracturable despite the increase in bone density, so main characteristic of the disease are is flimsy bones. Increased bone density causes limitation of bone marrow cavity that results in hematopoiesis failure and anemia or pancytopenia followed by hepatosplenomegaly and frontal bossing due to extra-medullary hematopoiesis. Overgrowth of bones can negatively affect patient’s movement and induce pressure on cranial nerve causing blindness, deafness or even facial paralysis and problem in swallowing. Unusual radiographic findings such as “diffuse sclerosis, bone within a bone” appearance and rugger-jersey spine can be detected in affected persons ^1–3^.

The disease has autosomal recessive, autosomal dominant and rarely x-linked forms. Autosomal Recessive Osteopetrosis (ARO), the most severe form of the disease has the incidence rate of 1 in 200,000 to 1 in 300,000. However, in some regions like Costa Rica its incidence is higher ^1^. Several genes have been revealed to be involved in ARO pathology, *TCIRG1* mutated in approximately 50% of cases, *CLCN7* in 10–15% of all ARO patients ^1^, *OSTM1* in 4%, five other genes in 5% of patients, and the remaining with unknown molecular pathology ^3^. After Aker’s study in 2013, mutations in *SNX10* were reported in eight ARO patients, and nowadays this gene is recognized to account for 4% of all patients affected by ARO ^3^. In the present study, a novel mutation is reported in this gene for a patient that analysis of *TCIRG1*, *CLCN7* and *OSTM1* genes did not detect any causative mutation.

## Case Report

**Figure 1.**
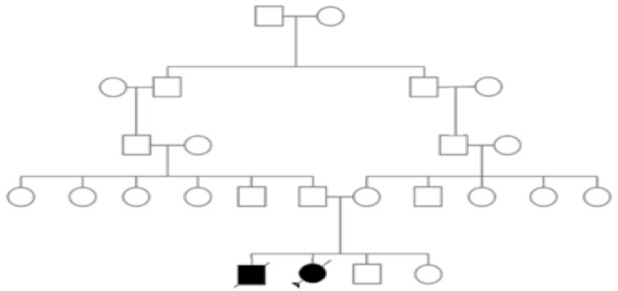
Pedigree of the investigated family.

A 15-year old girl was referred to us with possible diagnosis of osteopetrosis according to her clinical features. She had consanguineous parents. Her parents had another child even with more severe symptoms than her, who the patient and expired at the age of 6 years (Figure 1). This girl had problems in walking and reduced vision as the primary chief complaint in the first year of her life. She first experienced bone fracture when she was 3 years old and frequent fractures after that. She developed deformity in her limbs and limited movement due to this matter. Her vision problem developed to blindness. However, she had normal hearing. She had cranium deformity and mild hepatosplenomegaly. Laboratory investigations revealed intermediate anemia (average hemoglobin level: 9 *gr/dl*).

## Materials and Methods

### Samples

After obtaining informed consent from patient and her parents, blood samples were collected in EDTA tubes.

### Molecular studies

Genomic DNA was extracted using standard phenol chloroform method. The molecular analysis of genes *TCIRG1*, *CLCN7*, *OSTM1*, that are responsible for approximately 70% of autosomal recessive osteopetrosis cases, was performed by amplification and sequencing of all exons and intronexon boundaries. After the report that *SNX10* mutations can cause osteopetrosis, this gene was also included in the study. Primers for six exons of the gene were designed and synthesized (Pishgam Biotech Company, Tehran, Iran), with the sequence presented in table 1.

**Table 1.** Primers used for amplification of *SNX10* gene exons. Each amplified fragment contained the corresponding exon with at least 50nt of flanking introns

	**Sequence (5′->3′)**		**Length**	**Product length**
**Exon 1**				
	Forward primer	TCCAGCTTCCTCGCCAATTC	20	483
	Reverse primer	GGTGGGCCTTTGGTCTTTCA	20	
**Exon 2**				
	Forward primer	CTCCCACCTCAGTGTTGCAT	20	842
	Reverse primer	CCACGCAAGGCACATCATTT	20	
**Exon 3**				
	Forward primer	GGAGGTGTCTCTAAGCCCCA	20	733
	Reverse primer	AACATTTCTGAGGCCTTTCATGG	23	
**Exon 4**				
	Forward primer	CCAAAGTAATGCGTTGCTGG	20	698
	Reverse primer	AGCCACAAGATGGTGCTCTA	20	
**Exon 5**				
	Forward primer	AGTTAACATATGCTTTCCTCCCCT	24	790
	Reverse primer	CACAACACACTCAAAGCCTG	20	
**Exon 6**				
	Forward primer	ACACACACCTCCACACTGAA	20	748
	Reverse primer	TGGTAACACTGCCCCACTGA	20	

For the PCR reaction, the thermocycling conditions were: initial denaturation step at 94*°C* for 4 *min*, followed by 10 cycles of denaturation at 94*°C* for 30 *s*, annealing that decreased 1*°C* in each cycle from 70*°C* to 60*°C* prolonged for 30 *s* and amplification at 72*°C* for 50 *s*. After that, 25 cycles of denaturation at 94*°C* for 30 *s*, annealing at 60*°C* for 30 *s* and amplification at 72*°C* for 50 *s* and a final step of 72*°C* for 10 *min* was performed. Amplified fragments were checked with 1.5% agarose gel and visualized using safe DNA stain. The PCR products were purified by Expin Combo GP- Mini purification kit (GeneAll Biotechnology, Seoul, South Korea) and sequenced by ABI 3730xl automated sequencer (Pishgam Biotech Company, Tehran, Iran). The identified mutations were also confirmed by sequencing the opposite strand.

### Bioinformatics analysis

The sequence files were analyzed using available resources at the NCBI website, Sequence Scanner software v1.0 and Chromas software version 2.4.3. The effect of mutation was analyzed by multiple *in silico* tools including CADD and also Mutation taster.

## Results

### Molecular findings

**Figure 2.**
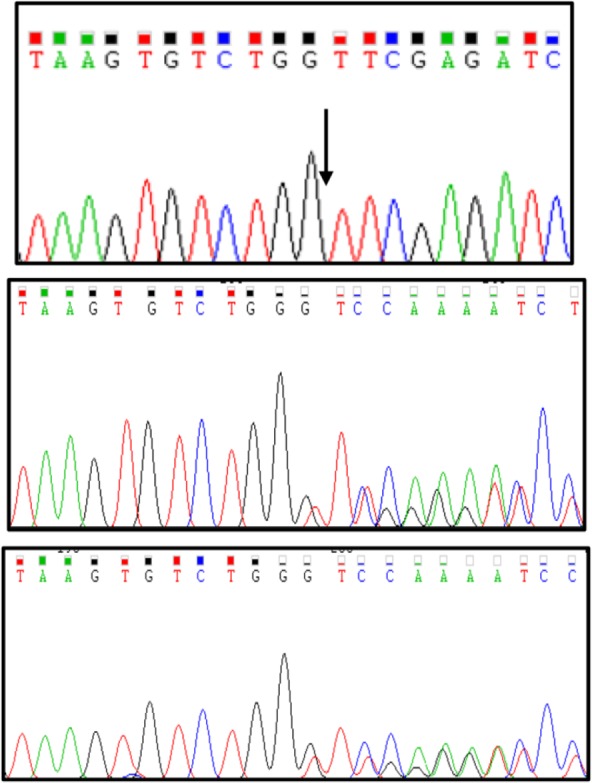
Sanger sequencing chromatograms showing the nucleotide deletion found in the patient in homozygous (Top panel; the arrow represents deletion of G between G and T) and in her parents in heterozygous states. Middle and bottom panels show her father and her mother sequences, respectively.

Based on the most frequent genes causing ARO, first molecular studies were performed on the genes *TCIRG1*, *CLCN7*, *OSTM1*, but the results revealed no mutation on these genes. Interestingly, sequence analysis of *SNX10* gene leads to identification of a homozygous variant (c.43delG) in exon 2 in the patient. Heterozygous status was confirmed in both of her parents (Figure 2).

### Bioinformatics analysis

Deletion of the G nucleotide leads to p.Val15Phe change at the deletion point and causes a frameshift which is ended with a premature termination of translation at 30th amino acid after the deletion point. Bioinformatics studies using CADD (phred score of 35 and raw score of 8.28) and Mutation taster (prediction: disease causing) lead us to infer this alteration as a pathogenic variant/mutation that can cause osteopetrosis.

## Discussion

In this study, an affected girl with osteopetrosis was introduced who had no mutation in most common causative genes (*TCIRG1*, *CLCN7* and *OSTM1*) for the disease, but analysis of *SNX10* gene was able to detect a novel homozygous deletion in her.

There are several types of osteopetrosis caused by different mutant genes ^1–3^. Homozygote mutations in *SNX10* gene, located on 7p15.2, causes autosomal recessive form of osteopetrosis. Translated protein from *SNX10* gene has 201 amino acids and belongs to Sorting Nexin (SNX) family that are cytoplasmic and membrane-associated proteins playing various roles in endocytosis and protein trafficking ^4^.

It has been revealed that SNX10 protein is involved in ciliogenesis ^5^. However, interestingly OPTB8 patients show no phenotype indicating dysfunction of cilia. Also, previous reports showed that protein can induce formation of giant vacuoles in many cell types ^6^. Additionally, SNX10 plays an essential role in vesicular trafficking. Since osteoclasts activity highly depends on this cellular phenomenon, mutation in *SNX10* gene can be easily assumed to cause osteoclast dysfunction and consequently osteopetrosis.

*SNX10*, like the other members of SNX family, has phox (PX) domain. PX domain is structurally conserved in eukaryotes and as a phosphoinositide-binding domain plays many different roles in cells such as cell signaling, vesicular trafficking, protein sorting and lipid modification ^7–9^. In SNX10 gene, this domain binds to phosphoinositide and mediates interaction between SNX10 and membrane trafficking. In our case, frameshift alteration causes production of a truncated protein which terminates 30 amino acids after mutation, that results in loss of most of PX domain residues spanning three phosphatidylinositol-3-phosphate binding sites and also ubiquitination sites of the protein. Therefore, it can be easily predicted that the shortened mutant protein is not able to have its native function in osteoclasts. Considering above evidences, we concluded that the novel variant c.43delG is the cause of osteopetrosis in the patient.

*SNX10* is responsible for less than 5% of ARO, but it appears that mutations in this gene show variable expressivity of clinical phenotype, even in the same family ^10^. This variability can be seen in the present study as well. Here we had an inbred family with two affected children. Their affected son expired at the age of 6 years; however, their affected girl lived for 17 years (approximately three times more than the boy). There is no evidence to show sex influence in ARO.

## Conclusion

In summary, we report a novel mutation of *SNX10* gene in a female patient with osteopetrosis who died at the age of 17 years. The family had an affected boy, apparently with more severe phenotype who died at the age of 6 years. Bioinformatics analysis strongly supported the pathogenic role for this mutation.

The role of *SNX10* mutations in osteopetrosis has been revealed not long ago. Analysis and reporting patients with mutation in this gene can be very helpful to obtain a better picture of the disease phenotype in SNX10-related osteopetrosis.
